# Targeting the SphK-S1P-SIPR Pathway as a Potential Therapeutic Approach for COVID-19

**DOI:** 10.3390/ijms21197189

**Published:** 2020-09-29

**Authors:** Eileen M McGowan, Nahal Haddadi, Najah T. Nassif, Yiguang Lin

**Affiliations:** 1 Guangdong Provincial Engineering Research Center for Esophageal Cancer Precise Therapy, Guangdong Pharmaceutical University, Guangzhou 510080, China; yiguang.Lin@uts.edu.au; 2 Central Laboratory, The First Affiliated Hospital of Guangdong Pharmaceutical University, Guangzhou 510080, China; 3 School of Life Sciences, University of Technology Sydney, Broadway, Sydney, NSW 2007, Australia; nahal.haddadi@gmail.com (N.H.); najah.nassif@uts.edu.au (N.T.N.)

**Keywords:** COVID-19, SARS-CoV-2, Sphingosine kinase, sphingosine-1-phosphate, G-protein-coupled receptors

## Abstract

The world is currently experiencing the worst health pandemic since the Spanish flu in 1918—the COVID-19 pandemic—caused by the coronavirus severe acute respiratory syndrome coronavirus 2 (SARS-CoV-2). This pandemic is the world’s third wake-up call this century. In 2003 and 2012, the world experienced two major coronavirus outbreaks, SARS-CoV-1 and Middle East Respiratory syndrome coronavirus (MERS-CoV), causing major respiratory tract infections. At present, there is neither a vaccine nor a cure for COVID-19. The severe COVID-19 symptoms of hyperinflammation, catastrophic damage to the vascular endothelium, thrombotic complications, septic shock, brain damage, acute disseminated encephalomyelitis (ADEM), and acute neurological and psychiatric complications are unprecedented. Many COVID-19 deaths result from the aftermath of hyperinflammatory complications, also referred to as the “cytokine storm syndrome”, endotheliitus and blood clotting, all with the potential to cause multiorgan dysfunction. The sphingolipid rheostat plays integral roles in viral replication, activation/modulation of the immune response, and importantly in maintaining vasculature integrity, with sphingosine 1 phosphate (S1P) and its cognate receptors (SIPRs: G-protein-coupled receptors) being key factors in vascular protection against endotheliitus. Hence, modulation of sphingosine kinase (SphK), S1P, and the S1P receptor pathway may provide significant beneficial effects towards counteracting the life-threatening, acute, and chronic complications associated with SARS-CoV-2 infection. This review provides a comprehensive overview of SARS-CoV-2 infection and disease, prospective vaccines, and current treatments. We then discuss the evidence supporting the targeting of SphK/S1P and S1P receptors in the repertoire of COVID-19 therapies to control viral replication and alleviate the known and emerging acute and chronic symptoms of COVID-19. Three clinical trials using FDA-approved sphingolipid-based drugs being repurposed and evaluated to help in alleviating COVID-19 symptoms are discussed.

## 1. Introduction

The novel betacoronavirus (2019-nCoV), also known as severe acute respiratory syndrome coronavirus 2 (SARS-CoV-2) [1,2] and pneumonia-associated respiratory syndrome (PARS) [3], causes coronavirus disease 19, or simply COVID-19. SARS-CoV-2 is rapidly evolving as the pestilence of the 21st century. As of September 2020, the number of COVID-19 cases has surpassed 33 million with nearly one million confirmed deaths worldwide. The incidence of COVID-19 continues to increase daily and has not shown any signs of abating (https://www.who.int/emergencies/diseases/novel-coronavirus-2019). Whilst approximately 80% of all cases will be either asymptomatic, or exhibit mild symptoms with no known ongoing health issues, 20% of patients will develop severe acute respiratory distress syndrome (ARDS), dyspnoea, low oxygen saturation, and lung infiltrates [4]. Complications of hyperinflammation, also referred to as the “cytokine storm syndrome”, are associated with the critical symptoms of the disease, including respiratory failure, septic shock, or multiorgan dysfunction, which, together, are the leading causes of morbidity and mortality in patients with severe COVID-19 [4,5]. More recently, it has come to light that a major complication of SARS-CoV-2 infection is the viral infectivity of endothelial cells lining the inside of blood vessels, causing major catastrophic damage to the endothelium, vascular dysfunction, and consequent multiple organ failure [6,7]. There is also a very high incidence of thrombotic complications in critically ill COVID-19 patients [8,9,10]. Additional severe complications of COVID-19 now include ischaemic stroke and brain damage, acute disseminated encephalomyelitis (ADEM) [11], and acute neurological and psychiatric issues [12].

Once the virus invades the host cell and replicates, the body will mount an inflammatory response to combat the excess manufacture of the virus particles. A major complication of viral infection is a hyperinflammatory response, characterised by exacerbation and dysregulation of the immune response and excess production of cytokines and chemokines during the acute phase of the illness (cytokine storm), leading to the attack and damage of not only infected cells, but normal cells as well [13]. In fact, it is this hyperinflammatory response that is the cause of many of the major health issues as well as increased mortality, particularly in elderly, immunocompromised, and comorbidity patients (such as those with diabetes and cardiovascular disease). One of the first studies to emerge from Wuhan, China, described patients with severe COVID-19 complications, admitted into the intensive care unit (ICU), as being more likely to present with higher plasma levels of inflammatory markers, including the interleukin 2, 7, and 10 (IL2,IL7, IL10), GMCSF, IP10, MCP1, MIP1A, and tumour necrosis factor alpha (TNFα) [14]. The association of the symptoms of hyperinflammation with disease severity and death was confirmed in other studies, strongly suggesting that the use of anti-inflammatory agents alongside antiviral treatments may reduce the rising mortality in severely compromised COVID-19 patients [15]. Recent pathology reports from deceased COVID-19 patients have shown severely compromised endothelium causing catastrophic multiorgan failure. Balancing the immune response to SARS-CoV-2 infection is critical for virus attenuation. Therefore, to combat the more severe effects of COVID-19, specifically ARDS, catastrophic vascular dysfunction, and secondary multiorgan failure, resulting from a hyperimmune response, “the plea” is for multitargeted interventions to trial combinational therapies and/or new targeted drugs [16].

The sphingolipid rheostat plays an important role in regulating viral replication, the innate, adaptive, and hyperinflammatory immune response, and importantly, maintaining vascular endothelial integrity [17,18]. Hence, targeting sphingosine kinase (SphK), sphingosine-1-phosphate (S1P), and the S1P cognate receptors (high-affinity G-protein-coupled receptors) in the repertoire of therapies to control viral replication, hyperinflammation, and aid in the maintenance of vascular endothelial integrity is highly attractive.

This review provides a comprehensive overview of SARS-CoV-2 infection, the resultant severe symptoms caused by the virus, our current strategies for treatment, and importantly, discusses how we can we use our knowledge of the SphK/ S1P/S1PR pathway to design therapeutic strategies to provide some relief from, and to combat, the severe, unprecedented symptoms of COVID-19 and the chronic health problems emerging in an increasing number of patients. To date, three clinical trials using FDA-approved sphingolipid-based drugs being repurposed in COVID-19 treatment are in progress and will be discussed. 

## 2. Coronaviruses from Animals to Humans

The first human coronavirus (hCoV) describing symptoms of the common cold was documented over 50 years ago in the British Medical Journal by Tyrell and Bynoe [19]. Coronaviruses were isolated from humans in the early 1960s in both Britain and the United States [20] and were identified by their crown-like morphology [21]. Their virion is between 80 and 120 nm containing a positive single-stranded RNA genome (ranging from 26 to 32 kb in length) encoding four enzymes essential for the viral life cycle [21,22]. Human volunteer studies in the 1960s and 1970s showed that after coronavirus exposure, the virus multiplied superficially in the respiratory tract with symptoms of mild respiratory infection [20]. Although coronavirus was found to be common in humans, due to the lack of severity of disease symptoms, the study of hCoV was relatively lacklustre for over 50 years [23]. In contrast, CoV has been well studied in animals due to its contribution to the rich diversity of animal diseases in many species, where it is sometimes fatal [23,24]. 

Sadly, this viral pandemic is not unexpected. A wake-up call to the severity of coronavirus infections in humans came with the outbreak of the human severe acute respiratory syndrome (SARS) in 2003 [25,26], and later the emergence of the Middle East respiratory coronavirus (MERS-CoV) in 2012 [27,28], causing major clinical pathology, mainly characterised by fever, dyspnoea, lymphopenia, and respiratory tract infections. This year (2020), the world is experiencing the worst health pandemic since the Spanish flu in 1918 [29]—the SARS-CoV-2 or COVID-19 pandemic [30,31].

Coronaviruses have a unique strategy of replication. Studies of CoV pathogenicity in animals have provided a strong base in the understanding of the origin and biology of the human SARS-CoV [23] and now SARS-CoV-2. SARS-CoV and SARS-CoV-2 are zoonotic pathogens, believed to have been transmitted to humans from an animal source. The understanding is that the SARS coronaviruses derived from viral transmission between animals from different species housed in the same cages in live-animal wet markets in China [1]. Gradual deletions and mutations in the viral genome enabled the emergence of these new human coronaviruses, crossing the animal-to-human barrier [1]. Bats are the most likely primary host of the SARS epidemic with genomic sequence and phylogenetic studies revealing 79.6% identity between human SARS-CoV and bat-coronavirus and 96% sequence identity between hSARS-CoV-2 and bat-coronavirus [2,32]. Although the transmission intermediary host of SARS-CoV-2 to humans is not known, a suspected intermediary reservoir associated with the COVID-19 outbreak is the pangolin (bat–pangolin–human) [33], yet to be confirmed or retraced.

## 3. SARS-CoV-2 Infectivity, Symptoms, and Complications

### 3.1. SARS-CoV-2 Infectivity

The extremely virulent nature of SARS-CoV-2 and the uncertainties surrounding the complexities of the disease have facilitated a surge in research of the biology, infectivity, and symptoms of the COVID-19 disease. SARS-CoV-2 (2019-nCoV) is transmitted human-to-human via airborne droplets, direct contact, or surface contamination [34,35]. The genome sequence of the 2019-hCoV/ SARS-CoV-2 (GenBank No. MN908947) is enclosed in a lipid envelope in which are embedded transmembrane glycoprotein spikes protruding from the viral surface [22]. These protruding spiked glycoproteins bind to specific receptors on the host cells to promote viral entry. The human angiotensin-converting enzyme 2 (hACE2) receptor, essentially expressed in all tissues, including the lung, heart, kidney, gut, brain, and vascular endothelial cells, has been identified as the functional entry coreceptor for SARS-CoV-2 [22,36,37]. The SARS-CoV-2 also employs the cellular serine protease TMPRSS_2,_ to facilitate entry into the host cell [38]. The virus then uses the host cell machinery to replicate and subsequently shed viral copies. 

The severity of symptoms appears to be dependent on patient age and immune system status, with mortality more prevalent in the >70 age group and immunocompromised individuals [4]. Comorbidities, including hypertension, diabetes, chronic obstructive pulmonary disease (COPD), cardiovascular disease, cerebrovascular disease, and pre-existing respiratory disorders, contribute as major risk factors for COVID-19 patients [39,40,41]. Systemic disorders known to be associated with COVID-19 infections are not confined to fevers and respiratory-related issues. Other mild to severe side effects include headaches, loss of smell (affecting the olfactory bulb in the brain), fatigue, haemoptysis, hypoxemia, gastroenteritis and diarrhoea, dyspnoea (difficult or laboured breathing linked to pulmonary disease or heart failure), lymphopenia, acute cardiac injury and thrombosis, neurological disorders, and brain damage [4,8,9,10,42].

### 3.2. COVID-19 and Respiratory Failure 

Whilst COVID-19 disease characteristics are similar to the common cold and influenza, SARS-CoV-2 viral infection has a higher transmission rate [4]. The primary cause of COVID-19 deaths is respiratory failure, mainly in the elderly and patients with other underlying health problems [32,43,44]. SARS-CoV-2 attacks both the lower and upper airways, varying from mild respiratory disorders, ground-glass opacities (observed in subpleural regions of the lung cavities), through to pneumonia, RNAaemia, and ARDS [4,45]. 

In the early stages, the patient’s immune response is triggered to clear the infection with the local and systemic elevation of blood cytokine and chemokine levels observed. Replication of cytopathic viruses, such as SARS-CoV-2, causes host cell death, tissue injury, and initiates an inflammatory response. If the infection is not cleared, the body elicits a surge in proinflammatory cytokines (the cytokine storm) associated with disease severity and death [14]. Hyperinflammatory alveolar oedema, a decrease in lung compliance, and reduced oxygen capacity are symptomatic of ARDS. Although patients with severe illness have more of a propensity to develop blood clots, large and small blood clots are frequently observed and are a major complication of COVID-19 [10]. Blood clots in the lungs can restrict oxygenated blood flowing through the lungs, and in cases where COVID-19 patients develop critically low blood-oxygen readings, it may explain why, in some cases, ventilators do not help to prevent respiratory failure [10]. 

### 3.3. COVID-19 Pulmonary Vascular Disorders 

In the few studies of lung morphology of deceased COVID-19 patients, the lungs exhibited distinct pulmonary vascular changes [46]. In COVID-19 patients, specific pathogenic features of the lungs include severe endothelial injury with the presence of intracellular virus with disrupted endothelial cell membranes (endotheliitis) [6]; an accumulation of inflammatory cells with evidence of endothelial and inflammatory cell death [6]; widespread vascular thrombosis with microangiopathy and occlusion of alveolar capillaries; and significant new vessel growth through intussusceptive angiogenesis [46], which refers to the expansion and remodelling of the wall of the blood vessel, splitting one vessel into two [47]. This pulmonary pathobiology distinguishes COVID-19 from other equally severe respiratory diseases, such as influenza viral infections [46]. 

The question of whether COVID-19-endotheliitis is a direct consequence of SARS-CoV-2 infectivity and/or a consequence of endocytosis of the viral particles by the endothelial cells may be partly answered in the publication by Ackemann and colleagues [46]. The observation that the SARS-CoV-2 receptor, ACE2, was more abundant on the endothelial cells of COVID-19 patients compared to uninfected subjects suggests direct entry [46], although this does not exclude viral entry by endocytosis or entry due to endothelial dysfunction. Endothelial morphological changes, including loss of adhesion contact with the basal membrane, cell swelling, and disruption of intercellular junctions were consistent with intracellular SARS-CoV-2 infection, suggesting that direct intercellular viral effects contributed to endothelial injury as well as perivascular inflammation, infiltrating lymphocytes, and subsequent multiple organ failure [6,46]. Pulmonary complications from breaches in the vascular endothelium include tissue oedema (build-up of lung fluid), deregulation of inflammatory cell infiltration, and intravascular coagulation, which all contribute to severe lung damage [48].

### 3.4. COVID-19, the Heart and Cardiovascular System 

SARS-CoV-2 has demonstrated a specific tropism towards the cardiovascular system, being responsible for severe acute and chronic cardiac diseases. Acute cardiovascular-related pathologies including congestive heart failure and brain medullary cardiorespiratory dysfunction are among the more severe health problems associated with COVID-19 disease [14,40,49,50,51]. Although a damaging correlation between cardiovascular disease (CVD) and COVID-19 susceptibility is widely accepted, the underlying reasons and the acute and long-term cardiac consequences are unclear [52]. On the one hand, COVID-19 may be a risk factor for CVD development through indirect action, including infection in the lungs causing an oxygen deficiency resulting in cardiac ischaemia, and further activating the immune system associated with a pronounced cytokine storm [53]. Whilst early activation of the immune response is important in virus control, persistent severe systemic inflammation (immune activation of cytokines, such as IL6 and TNF) and the release of troponin and natriuretic peptides, especially in predisposed patients (as present in immunocompromised patients, the elderly, and those with comorbidities such as diabetes and CVD) can result in organ failure. Inflammation can result in diffuse microangiopathy with thrombosis in the vascular system, a symptom observed in the pulmonary arteries in the lung pathology of COVID-19 patients. Myocarditis, heart failure, acute coronary syndrome, and cardiac arrhythmias, also resulting from acute inflammatory responses, lead to rapid deterioration and death [51]. On the other hand, it is undisputed that patients with comorbidities are more vulnerable to cardiac complications if they succumb to COVID-19 [54]. Patients with pre-existing CVD and hypertension are widely representative of poorer prognosis and have a higher risk of dying due to SARS-CoV-2 infection [51,55]. The ACE2 receptor, which is responsible for SARS-CoV-2 entry into host cells, is highly expressed in perivascular pericytes and cardiomyocytes. The suggestion is that direct infection of the cardiac cells may lead to myocardial, endothelial, and microvascular dysfunction and myocardial infarction [49,51]. However, ACE2 has well-documented cardioprotective effects, important in the regulation of the renin–angiotensin–aldosterone system (RAAS), influencing vasculature and blood pressure. Conversely, ACE2 is also upregulated in the failing human heart [49,52]. Therefore, whether the ACE2 receptor can be used to target therapy for COVID-19 patients, especially patients with underlying heart problems, is debatable [40,49,50,51,52,53,56]. As mentioned, the ACE2 receptor is also highly expressed on the surface of endothelial cells of COVID-19 patients, and an important specific pathogenic feature of COVID-19 patients is endotheliitis [6]. Endothelial dysfunction is also linked to the failing heart circulation and plays a vital role in the development and progression of CVD and heart failure [57], and needs to be addressed in any severe COVID-19 patient treatment plan. In COVID-19 treatments, it is important to keep in balance the principal pharmacological interactions between the drugs used in cardiovascular disease and the treatments and potential vaccines for COVID-19 patients.

### 3.5. Vascular Endotheliitis Contribution to Multiorgan Failure in COVID-19 Patients 

Disruption of the vascular endothelium by the invasion of SARS-CoV-2 may lie at the core of multiorgan failure [6,58]. The vascular endothelial lining forms a protective barrier around blood vessels (arteries and veins), including the lining of the heart chambers and the lymphatic vessels [59]. This layer of endothelial cells is essential in the maintenance of vascular homeostasis, vascular integrity, and barrier function [59]. Endothelial cells line the surface of the entire circulatory system and contain the ACE2 receptor, making them directly vulnerable to SARS-CoV-2 virus invasion [6]. Although the major overt symptom of COVID-19 mortality is pneumonia, linking the SARS-CoV-2 pandemic to a classic respiratory disease, one of the first critical adverse symptoms observed in many patients was major blood vessel damage in the lung pathology after death. The invasion of SARS-CoV-2 into the endothelial cells throughout the vascular endothelium, or COVID-19-endotheliitis, may explain the systematic impaired microcirculatory function, leading to devastating multiorgan failure [60]. Around 20–30% of severe COVID-19 patients develop a “storm” of blood clots throughout their bodies [10]. The integrity of the vascular endothelial lining helps to prevent blood clot formation. The development of the COVID-19-related thromboembolic phenomenon (the higher frequency of small and large blood clots) in severely ill COVID-19 patients may, in part, be due to damage of the endothelial cells by SARS-CoV-2 infectivity, releasing proteins that trigger unusual clot formation into the blood vessels [10]. Damaged blood vessels are more susceptible to other opportunistic infections, potentially causing septicaemia (blood poisoning), which can also lead to multiorgan failure. Maintaining vascular endothelium integrity is key in controlling the immune response, and this links with the critical role of SphK-S1P-S1PR in the control of endothelium integrity, which will be explored in a later section. 

### 3.6. Neurological Disease and COVID-19 

Coronaviruses are historically found to be neurotropic [61]. The majority of COVID-19 patients experience mild neurological symptoms such as headaches and loss of smell, neurological symptoms that suggest the coronavirus is affecting the brain [62]. More serious neurological symptoms experienced by COVID-19 patients range from encephalitis, encephalopathies, acute disseminated encephalomyelitis and myelitis, cerebrovascular disease and seizures, ischaemic strokes, intracerebral haemorrhages, and altered mental health status [63]. Recently, there is an unusual increase in cerebral venous thrombosis in both young and old COVID-19 patients whereby thrombotic occlusions cause a high risk of acute ischaemic strokes [64,65,66].

The cerebrovascular endothelium is critical in maintaining blood vessel integrity and cerebrovascular homeostasis. As mentioned, disruption of the vascular endothelial barrier and ensuing hyperinflammation are key features of COVID-19. Inflammation and loss of endothelial integrity contribute to brain oedema and poststroke neuronal injury. Earlier evidence supports SARS-CoV direct invasion of the central nervous system (CNS), as reported in the brains of patients and in animal models, and spread of the virus through the synapse-connected route to the medullary cardiorespiratory centre [62]. The ACE2 receptor, required for SARS-CoV-2 cell entry, is present in brain vascular endothelium, and could facilitate direct viral entry into the host brain cells [63]. There are also a number of potential routes of SARS-CoV-2 entry into the brain that may cause direct damage including through the unprotected olfactory bulb, carriage across the blood–brain barrier (BBB) following viraemia, or by infected leukocytes. Alternatively, brain damage may be a result of hyperinflammation due to the body’s immune response (innate and adaptive) [63]. Recently, a probable case of Parkinson’s disease after SARS-CoV-2 infection has been reported with the suggestion of a causal association between SARS-CoV-2 infection and Parkinson’s disease [67]. Hence, monitoring of all COVID-19 patients for stroke [66,68] and potentially Parkinson’s, as well as early intervention, is recommended [67], especially with the strong possibility that “silent” brain damage caused by SARS-CoV-2 may be a price we pay later, and perhaps many years later.

### 3.7. Rare Inflammatory Diseases Associated with COVID-19 

The current viewpoint is COVID-19 is mainly asymptomatic or shows mild symptoms with a better prognosis in most children [69,70]. Two rare inflammatory diseases, Kawasaki disease and a new inflammatory disease, named “paediatric inflammatory multisystem syndrome temporally associated with SARS-CoV-2” (PIMS-TS), are both causally linked to COVID-19. Without any clear scientific evidence, the prevalence of both of these rare diseases has increased following infection by SARS-CoVs [71,72,73,74]. Kawasaki disease causes the blood vessels to become inflamed due to over-reaction of the immune system, with symptoms including fever, rash, bloodshot eyes, and joint pain [75]. Although symptoms for PIMS-TS are similar to Kawasaki, patients present with signs of toxic shock syndrome with severe abdominal pain, persistent fever, oxygen deprivation, rash, and conjunctivitis, with some children developing inflammation of the heart [71,72]. COVID-19 is speculatively emerging as a possible contributor to PIMS-TS symptoms in children [71]. 

### 3.8. The Cytokine Storm (Hyperinflammation, Morbidity and Mortality) 

Inflammation, in general, is a beneficial and highly regulated reaction to any hazardous insult to the body, such as in response to a pathogen to eliminate any impending threat and injury, and to repair any damage to restore and maintain homeostasis [76,77]. With acute viral overload, the immune response goes into overdrive, resulting in hyperinflammation, producing excessive cytokines. The “cytokine storm syndrome” is an important, consistent, and central theme in COVID-19 literature [13,78,79]. Understanding the mechanisms underlying the COVID-19-specific cytokine storm and targeting and quelling the hyperinflammatory response is necessary for the prevention of severe morbidity and death in the current COVID-19 pandemic, and also in any future viral outbreaks. This hyperinflammatory “out of control” immune response is common in many types of infectious—viral, bacterial, and non-infectious—diseases, cancer, and any flares in immunoinflammatory diseases, and is not COVID-19 specific [80]. Although there are many similarities, there are, however, subtle differences in COVID-19 infections that need to be taken into consideration. Without an understanding of the full complexity of COVID-19 disease, alleviating the symptoms of the cytokine storm with current drugs may not be sufficient, and may, in some cases, be detrimental. As previously briefly mentioned, there are specific vasculature pathobiological features that distinguish COVID-19 from other equally severe respiratory diseases, such as influenza viral infections [46]. These are mainly associated with atypical changes in endothelial morphology and functionality [6,46]. Therefore, maintaining a balance between a beneficial immune response to enable viral elimination and a hyperinflammatory response, which in most cases is detrimental, may be a matter of timing in therapy regimes. We will later discuss how SphK/S1P plays a vital role in endothelial homeostasis and the regulation of inflammation in pathogenicity, and how we can potentially target specific components of this pathway in adjunct viral treatments.

## 4. Current Strategies for the Prevention and Treatment of COVID-19 

### 4.1. COVID-19—Vaccine Development Targets

No vaccine or specific antiviral therapy for human-infecting coronaviruses have been approved to date. Herd COVID-19 prevention by vaccination is on the horizon but with months (and possibly years) to go before any approval [81,82]. The average time required to develop a vaccine is 2–5 years. Once a vaccine becomes available, it needs to undergo extensive preclinical trials with testing in cell culture and animals, from phases I to IV human clinical trials to the safety of use and efficacy, and then approval for use by the appropriate regulatory bodies, followed by mass production and ensuring global access [83]. Vaccines need to be safe for human use and accessible to all countries for world immunity to control the pandemic. 

COVID-19 vaccine development includes: (i) inactivated or live attenuated virus, (ii) subunit vaccines, which use part of the virus to mount an immune response, (iii) genetic vaccines (delivering messenger RNA to cells to counteract the virus RNA), (iv) viral vector vaccines engineered to produce part of the viral protein to mount an immune response, and (v) targeting the viral reproductive machinery [84,85]. 

Vaccine targets include antibodies to prevent SARS-CoV-2 host cell entry [38]. The coreceptors ACE2 and TMPRSS_2_ are prime neutralising antibody targets for blocking SARS-CoV-2 entry into host cells to stem host infectivity [22,38]. Although these target receptors sound promising, we do not fully understand the efficacy and adverse side effects of blocking these receptors, especially on the heart where they mediate important functions in the regulation of RAAS [38,49,52,86], and the fact ACE2 receptors are found on the endothelial cells lining the vascular circulatory system. Molecularly targeted therapies in the pipeline will still need extensive testing before clinical use. 

### 4.2. Current COVID-19 Vaccine Clinical Trials 

The ongoing marathon to develop the ideal COVID-19 vaccine is one of the world’s biggest challenges to date and is constantly evolving by the day. There are hundreds of vaccines currently being developed worldwide and a few promising trials are briefly described. When starting this review, scientists at the Jenner Institute, Oxford University, ChAdOx1 nCoV-19, had just developed a genetically altered adenovirus which causes a common cold in chimpanzees that cannot grow in humans; now, it is undergoing the final stages of stage III clinical trials (ClinicalTrials.gov Identifier NCT04324606). The ChAdOx1 nCoV-19 vaccine expresses a spiked glycoprotein, which is the protein enabling the SARS-CoV-2 to enter human cells via the ACE2 receptor. Hopefully, by recognising this spiked protein on the ChAdOx1 nCoV-19 vaccine, the body will develop an immune response and help to prevent viral entry into human host cells. However, clinical trials have been questioned due to an adverse reaction to the ChAdOx1 nCoV-19 vaccine (AstraZeneca). Recent developments also include “molecular clamp” technology, which clamps the viral glycoprotein spike in shape for immune recognition and neutralisation, and clinical trials of this technology commenced in Australia on 13 July 2020 [87]. Another promising COVID-19 clinical trial (BRACE, identifier NCT04327206), spearheaded by the Australian Murdoch Children’ Research Institute (MCRI) Melbourne, is using the bacille Calmette–Guerin (BCG) vaccine. BCG is a vaccine against tuberculosis, not a specific COVID-19 vaccine, and aims to boost the host’s innate immune system to reduce the prevalence and severity of SARS-CoV-2 infectivity. The antiviral drug, remdesivir, a nucleotide analogue that inhibits viral RNA polymerases [88], has been shown to reduce COVID-19 symptoms and is currently in limited clinical use. An interim analysis of the Adaptive COVID-19 Treatment Trial (ACTT) found remdesivir aids recovery 31% faster than a placebo, with approximately 3.6% benefit in survival [85,89]; however, there are concerns with liver damage in some patients [90]. Other promising vaccines are: mRNA1273 (Moderna therapeutics; Clinical trial identifier, NCT04283461); BNT162b2 (Pfizer; Clinical trial identifier, NCT04380701); CoronaVac (Sinovac; Clinical trial identifier, NCT04383574); Ad5-nCoV (CanSino Biologics; Clinical trial identifier, NCT04313127); Sputnik V/Gam-COVID-Vac (The Gamaleya National Center of Epidemiology and Microbiology: Clinical trial identifier, NCT04436471). It is envisaged that an antiviral vaccine alone will not beat this disease and combinational therapy will still be required for control and prevention. 

### 4.3. COVID-19 Vaccine Limitations 

Limitations in the development of a COVID-19 vaccine include the number of COVID-19 trial vaccine recipients who prove to be resistant to SARS-CoV-2 infection and monitoring of the adverse vaccine side effects. Even in the event of a successful vaccine, it is highly likely a vaccine would be required every year and would have to be revisited due to the prevalence of viral mutations. A vaccine would most likely kerb SARS-CoV-2 spread, meaning there will still be a need to find a drug that will reduce the severity of the COVID-19 symptoms. Given the virulent nature of SARS-CoV-2 and the urgency of curbing the pandemic, fast-track repurposing of known drugs is an attractive proposition to stem the pandemic and circumvent any complications associated with the development of new vaccines and anti-SARS-CoV-2 drugs [91]. 

### 4.4. Current COVID-19 Treatment 

In 1918, the flu pandemic was controlled by distancing, strict hygiene, quarantine, surveillance of communities, and notification of suspected cases [92]. Over 100 years later, in the absence of a vaccine, sadly, current prevention of SARS-CoV-2 infections has not changed; rather, these methods have been advanced by the technology of contact tracing. Early detection and quarantine of suspected and confirmed COVID-19 patients, early diagnosis, and supportive treatment, including oxygen therapy and antibacterial drugs to prevent pneumonia, are the most effective measures available. Current treatments include the management of symptoms alongside repurposing of such nonspecific antiviral agents, corticosteroids, herbal drugs, and the controversial antimalarial drugs [93]. Most COVID-19 patients will recover from the disease with standard care; however, immunocompromised patients, elderly patients with comorbidities, and vulnerable children (with undefined predisposition), are more likely to develop a hyperinflammatory response and need intubation and protective mechanical ventilator support to alleviate symptoms [40,49,50,51,52,53,56]. Severely affected COVID-19 patients are more likely to die from acute respiratory failure (ARDS), including pulmonary oedema or cardiovascular problems, causing acute myocarditis and inflammation of the heart muscle. The chronic and more long-term effects of COVID-19 are unknown making treatment difficult to manage.

#### COVID-19 Treatment—Targeting Vasculature Failure

As recently highlighted, vasculature failure may be one of the major underlying causes of COVID-19 mortality [6,46]. Approximately 40% of COVID-19-related deaths are due to cardiovascular complications, supporting that COVID-19 is looking more like a vascular infection in preference to a purely respiratory problem. Multiorgan inflammation, including the lungs, ear, kidneys, liver, bowel, and multiple severe blood clots, suggested impeded blood flow and the proposition that COVID-19 was also a vasculature sickness. Therefore, the theory is that conventional antiviral therapies are insufficient and potentially a drug that stabilises the vascular endothelium may be a more effective antiviral therapy for COVID-19 patients. The concept of using antistatins to reduce cardiovascular complications, thrombotic events, and inflammation as a protection and to mitigate endothelial dysfunction due to SARS-CoV-2 infection is currently being considered [94]. However, treatments with statins for COVID-19 patients come with some controversy, one being that statins are known to upregulate the ACE2 receptor (the receptor for SARS-CoV-2), therefore the jury is still out [94,95,96]. 

Although anti-inflammatory agents would not routinely be recommended for SARS-CoV-2 patients, the timely intervention of nonspecific anti-inflammatory and immunosuppressive drugs, alongside antiviral drugs, oxygen therapy, intubation, and protective mechanical ventilator support, has been proposed to alleviate symptoms for severely affected COVID-19 patients to reduce mortality [15,97]. Curbing hyperinflammation is especially relevant for COVID-19 patients with pulmonary oedema and hyaline membrane formation to prevent ARDS development and ongoing chronic problems. 

## 5. Targeting SphK/S1P/S1PR in Viral Infection and Alleviation of COVID-19 Symptoms 

A proven vaccine or treatment to suppress the severity of COVID-19 disease and reduce morbidity and mortality is not available (as discussed in Section 4 above). This multifaceted complex disease needs a systemic and systematic approach to block viral replication and alleviate the acute and chronic symptoms of COVID-19 disease (as overviewed in Section 3). Sphingolipid signalling plays integral roles in viral replication, activation of the immune response, and importantly, in maintaining vasculature integrity. Modulation of sphingolipid signalling has demonstrated many beneficial effects, including limiting inflammation, selective vascular barrier protection, regulating blood coagulation, cardioprotection, and neuroprotection to name a few of the COVID-19 symptoms. Hence, selective targeting of SphK-S1P-SIPRs to alleviate the acute and chronic effects of SARS-CoV-2 infection, as anti-COVID-19 adjunct therapies is worth considering. The complexity of SphK-S1P signalling and the seemingly opposing actions of SphK and S1P intra- and extracellular signalling on viral infection outcomes are discussed in more detail in this section. FDA-approved sphingolipid-based drugs are already available for immune-based diseases, and repurposing of these drugs to treat COVID-19 disease is on the cards with three FDA-approved sphingolipid-based drugs currently in clinical trials (reviewed in more detail in Section 6).

### 5.1. The Sphingosine Kinase Rheostat 

**Figure 1:**
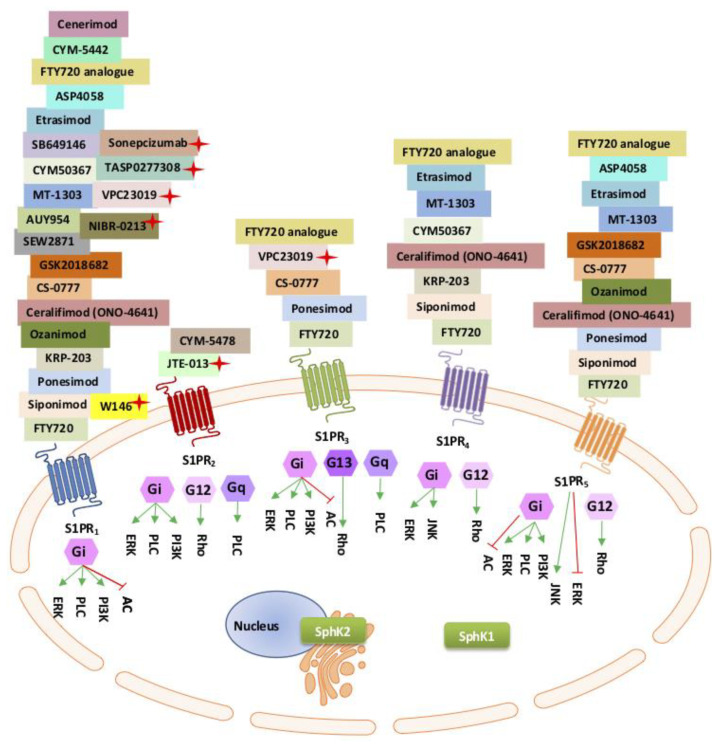
**Schematic summary of Sphingosine 1 Phosphate (S1P)/S1PR (G-protein-coupled receptor) agonist and antagonist signalling.** There are five S1PR (G-protein-coupled receptors) differentially expressed depending on cell type. Diversity of intracellular signalling is dependent on the differential targeting and binding of S1P to the S1P receptors, which results in internalisation of the S1P receptor and Gi-mediated downstream activation of effector pathways. S1P agonists and antagonists (described in Table 1) bind to cognate transmembrane S1PRs on the cell and are designed to block or activate S1PR action. S1P antagonists are depicted with the symbol. Adapted from [102,115].

Sphingolipids are ubiquitous key components of the lipid membrane and act as signal transduction molecules inside and outside of the cell [98,99,100]. Sphingosine is one of the sphingolipids important in these cellular processes. Sphingosine is produced by the action of ceramidase on ceramide in the sphingolipid pathway [101]. The phosphorylation of sphingosine to its active form, S1P, is catalysed by the SphK isozymes (SphK1 and SphK2) and their isoforms [100,101,102,103]. In turn, S1P is dephosphorylated by S1P lyase as part of the sphingolipid (SphK/S1P) rheostat. In its phosphorylated form, S1P acts as an intra- and extracellular messenger contributing to cellular signalling cascades and pathological processes referred to as an “outside–inside” S1P signalling, or paracrine/autocrine, mechanism of action [98,99,104]. The isoenzyme SphK1 has also been found to be released from the cell and contributes to S1P synthesis in the extracellular environment, as well as internal, autocrine activation [104,105]. Signalling by secreted S1P is mediated mainly through a family of transmembrane G protein-coupled receptors (GCPRs), designed as EDG isoforms or S1P receptors (S1PR_1-5_) [106,107,108]. However, both intra- and extracellular S1P action can occur independently of its cognate receptors [98,99]. Different S1PRs are expressed on various cell and tissue types and each of the S1PRs are coupled to different G-alpha subunits, extending the diversity of intracellular signalling processes in normal development and pathogenicity (Figure 1) [109,110,111]. Of the five S1P receptors (S1PR_1-5_), S1PR_1-3_ are expressed predominantly in vascular endothelium, the central nervous system (CNS), and the immune system, S1PR_4_ expression predominates in lymphoid tissue, and S1PR_5_ is found mostly in the CNS, immune natural killer cells and spleen [112,113,114]. 

The pleiotropic nature of S1P-S1PR paracrine signalling enables a wide range of physiological functions. Paracrine plasma-S1P in vascular endothelium and the lymphatic system is well characterised and known to maintain homeostasis and confer protection against vascular barrier dysfunction. 

### 5.2. SphK-S1P-S1PR_1_ Autocrine and Paracrine Inflammatory Actions

SphK/S1P signalling in innate and adaptive immune responses, both in immune trafficking and activation, is well-recognised and S1P/S1PRs are important mediators in multiple immune disorders [116,117]. Depending on the cellular environment, SphK-S1P action can be either pro- or anti-inflammatory [116,118]. This conundrum was observed in SphK1 and SphK2 knockout mice, whereby some studies demonstrated reduced colonic and synovial inflammation in TNF-induced arthritis [119] and others reported normal acute and chronic inflammatory responses [120]. Autocrine action of SphKs is believed to play a critical role in the regulation of S1P inflammatory innate and adaptive immune responses in inflammatory diseases, and in response to pathogens. There is some evidence in the literature that intracellular SphK1 might be considered proinflammatory and SphK2 considered anti-inflammatory based on SphK1- and SphK-null mice studies [121]. However, as demonstrated in SphK1-null and SphK2-null mice, there is some redundancy in function, obscuring the distinct functions of the two SphK isozymes [122]. The subcellular localisation of the individual SphK isozymes, as well as the locations of the S1PRs, which vary with tissue type, influence S1P activity, and the distinct, individual, and common, roles they play in viral pathogenesis, and inflammation in particular. 

### 5.3. Autocrine SphK-S1P Response in Systemic Inflammation and the Immune Response

The immune response is the body’s main protection against viruses; however, with SARS-CoV-2 infection, there appears to be a critical point where the immune response alters from a protective response to a destructive hyperinflammatory response [123]. Therefore, understanding the immunopathogenesis of COVID-19 and blocking hyperinflammation (cytokine storm) is critical for effective therapies. 

Cytokine responses are vital to evoke a host defence against pathogens [124]. The first line of defence, the innate immune response, needs to be able to detect and block pathogen infectivity. Individuals with an effective innate immune response recover faster when infected with a novel virus. Inflammatory cytokines such as interferons (IFNs) and tumour necrosis factor alpha (TNFα) have evolved to mediate viral tropism in both a positive and negative manner at different levels of infection [124]. In 1998, Xia and colleagues demonstrated that the pleiotropic cytokine TNFα induces intracellular SphK1 and activation of S1P to mediate endothelial cell activation, and adhesion molecule expression, thereby providing a mechanism for endothelial cell activation during systemic inflammation and the immune response [125]. Intracellular activation of SphK1 also associates with the TNF-receptor-associated factor 2 (TRAF2) and generates S1P locally, within the cell, mediating TNFα-stimulated nuclear factor kappa B (NFkB), promoting cell survival and proinflammatory mediators [126,127]. SphK1 activation is also important for lipopolysaccharide (LPS)-induced IL6 production and is implicated in exacerbation of inflammatory responses [128]. Intracellular generation of S1P in the cytoplasmic has also been shown to function as an epigenetic coregulator in LPS-induced lung inflammation [129].

Thus SphK1-S1P has two seemingly opposing modes of action (the yin and yang of SphK1 action). In response to infection, SphK1 activation and the export of S1P have a positive effect in protecting vascular endothelial barrier function (paracrine effect), whilst intracellular SphK1-S1P signalling stimulates proinflammatory cytokine release (autocrine effect) in the fight against infection. Excess cytokine release, contributed by SphK/S1P signalling, has the potential to contribute to detrimental hyperinflammatory responses or the cytokine storm. 

Similarly to SphK1, SphK2 has the same capacity to phosphorylate sphingosine to S1P, therefore teasing out and interpreting the distinct downstream functions of the two isozymes has been, and still is, difficult. Differing subcellular localisations within the cells and different kinetic properties provide distinct, as well as compensatory and redundant, signalling events [102,103]. SphK2, known to be located in the nucleus, endoplasmic reticulum, and mitochondria, is mainly associated with intracellular S1P activation, whereas SphK1, located mainly in the cytoplasm, is associated with S1P “outside–inside” events. SphK2 has been linked to both pro- and anti-inflammatory responses. From observations in SphK2-deficient mice (SphK2-/-), SphK2 was believed to be a negative regulator of inflammation [121]. However, in the SphK2-/- mouse, the elevation of SphK1-S1P expression compensated for the lack of SphK2, thus blurring the true effect of SphK2 activation [121]. Evidence for the proinflammatory effects of SphK2 includes SphK2-S1P regulation of histone acetylation (HDAC-1/2) and the transcription of proinflammatory genes in the nucleus, thus promoting the inflammatory response [130,131]. 

Alternatively, SphK2 has been shown to have an anti-inflammatory response in human macrophages, which are the main regulators of inflammation [132]. In the experiments conducted by Weigert et al., overexpression of SphK2 suppressed NF-κB transcriptional activity and cytokine release [132]. Early degradation of SphK2 was shown to elicit macrophage activation, and late upregulation of SphK2 may be involved in terminating inflammatory cytokine production; however, it is important to note that these actions were shown to be independent of S1P activity, as demonstrated by using a SphK2 kinase-dead mutant [132]. Thus, modulation of S1P activity, either through SphK1 or SphK2 or the specific S1P lyase, would be a novel therapeutic approach in inflammatory control.

The use of specific inhibitors or inhibitors that preferentially target SphK1 and/or SphK2, are currently in use, as are developments to help delineate the functions of each SphK isozyme (Table 2) [133]. In addition, inhibitors of S1P and/or S1PR_1-5_, are also being evaluated (Figure 1 and Table 1) and developed for clinical application in multiple complex diseases including autoimmune diseases, chronic inflammatory diseases, diabetes, and multiple organ failure [102,110,134]. 

### 5.4. The S1P/S1PR Paracrine “Outside–Inside” Response 

Here, we refer to the “*outside–inside*” response specifically related to extracellular S1P binding to transmembrane proteins, the S1PRs or G-protein-coupled receptors, on the outer cell surface, acting as an intracellular second messenger, to activate a wave of intracellular signalling events, (Figure 1) [178]. 

In response to external stimuli, including proinflammatory cytokines, SphK1 translocates to the plasma membrane, close to the sites of sphingosine production and localisation, resulting in a transitory elevation of S1P expression. S1P is then exported by specific transporters into the extracellular environment as an extracellular first messenger [18,206,207]. S1P can, in turn, activate cognate receptors (S1PR_1-5_) on neighbouring cells and self, acting in a paracrine manner [18]. S1P binding to S1P receptors differentially regulates the innate immune response, as reviewed extensively by Bryan et al. [117]. Plasma S1P is carried in the bloodstream by albumin or the high-density lipoprotein (HDL)-apolipoprotein M (ApoM) [208,209]. There is evidential support for albumin-bound-S1P to exert different physiological responses compared to S1P bound to HDL-ApoM, whereby high levels of HDL-ApoM-S1P have a protective role in vascular and endothelial function [208,209]. 

ApoM carries S1P preferentially to S1PR_1,3_ and induces S1PR internalisation and Gi downstream activation of effector pathways [208,209] (Figure 1). Many of the intracellular second messenger signalling pathways remain obscure and somewhat controversial. The most studied S1P outside–inside S1P receptor pathway is the S1P/S1PR_1_ pathway signalling. Extracellular S1P complexes with S1PR_1_ forming complexes with ß-arrestin to regulate intracellular effector pathways, including tyrosine kinase signalling (extracellular signal-regulated kinase-1/2 [ERK-1/2]), the platelet-derived growth factor receptor ß (PDGFRß), and the PKB/mammalian target of rapamycin (mTOR pathway) [210]. Thus, S1P/S1PR_1_ intracellular signalling is believed to control various biological responses including growth, differentiation, cell migration and trafficking, and in addition, pathological inflammatory responses. 

Development of S1PR agonists and antagonists are of interest to the pharmaceutical industry, due to their high potential in the treatment of immune-mediated diseases and cancer. Table 1 provides a comparative list of the S1P modulators, which are currently in use to understand the biology of the S1PRs as well as their usefulness as therapeutics.

### 5.5. The Sphingolipid Pathway in Coronavirus Infection and Replication

Enveloped RNA viruses, such as SARS-CoVs are very much dependent on the host’s lipid biosynthesis, and there are a growing number of examples in which the sphingolipid pathway and SphK/S1P intracellular signalling has been shown to play an integral role in viral permissiveness and replication [101,211,212,213,214,215]. Viruses exploit the host cell metabolism in all stages of their life cycle with the sphingolipid rheostat central in this process [101]. Sphingolipids serve as coreceptors during viral entry, modulate the virus replication cycle, and influence the antiviral immune response [216]. Cells overexpressing SphK1 are more susceptible to viral infection with increased virus replication and produce more virus proteins than the control cells. SphK2 colocalise with viral RNA and blocking SphK2 significantly impairs viral function [133,183].

Viral infection leads to rearrangement of cellular membranes and manipulation of the lipid metabolism to support viral entry and virus particle production. Viruses manipulate cellular signalling. Examples of viral exploitation of SphK/S1P are influenza [217], measles [213], hepatitis B [218], dengue virus [219], the respiratory syncytial virus (RSV), hepatitis B virus (HBV), and hepatitis C viruses (HCV) [220]. 

The influenza virus is a good example of an enveloped virus taking advantage of the host sphingolipid metabolism in the process of replication and to modulate the host defence system. Influenza infection increases intracellular SphK1-S1P and SphK-activated signalling pathways such as (1) NF-kB and ERK and (2) MAPK and PI3K/AKT, which are necessary for viral protein synthesis, the amplification of progeny virus, and nuclear export and transport of viral ribonucleoprotein (RNP) complexes to facilitate the production of infectious viral particles, respectively [212,221]. Conversely, blocking SphK or S1P lyase, the enzyme that irreversibly converts S1P back to sphingosine; inhibits influenza virus proteins; interferes with the ERK-AKT pathway; blocks the activation of Ran-binding protein 3 (RanBP3), a cofactor for chromosome region maintenance (CRM1); and inhibits the CRM1-mediated nuclear viral export of infectious progeny viruses [211,212]. Thus, inhibition of SphK1 was shown to impair the influenza viral life cycle. Similarly, the measles virus (MV), an enveloped RNA virus, was also shown to manipulate SphK-S1P signalling by inducing a transient increase in S1P, activating the metabolic mTORC_1_ pathway, and heat shock protein 90 (hsp90), which are vital for efficient MV replication [222]. Virus uptake was not affected by the SphK inhibitor, SKI-II, and the ceramidase inhibitor, ceranib-2; however, impairment of mTORC1 and HsP90 created a hostile environment for viral replication [222]. These experiments were conducted in primary human peripheral blood lymphocytes and human B cells [222]. Inhibitors of intracellular SphK1/S1P may have a place in adjunct therapy in restricting viral replication and viral load in SARS-CoV-2 in the current SARS-CoV-2 pandemic.

### 5.6. The S1P/S1PR_1_ Response to Inflammatory Lung Viral Infections

Once SARS-CoV-2 enters the chest it can cause inflammation, pneumonia, ARDS, and sepsis complications with irreparable damage to the lungs. There is no specific treatment; however, in extreme cases, mechanical ventilation is the only option. Extended use of mechanical ventilation in the treatment of the more severe COVID-19 respiratory problems is associated with ventilator-induced lung injury (VILI), characterised by loss of alveolar permeability and the influx of inflammatory cytokines contributing to excessive lung stress and increased morbidity [223]. In a study by Suryadevara et al., using a well-characterised VILI mouse model, S1P lyase inhibition, thus increasing S1P levels, demonstrated a protective role for SphK1 and S1P against VILI [224]. Hence, patients suffering from lung damage, from viral hyperinflammation or mechanical injury, may have some therapeutic benefit by restoring S1P levels, either by increasing production through SphK1 or by preventing the degradation of S1P.

Previous reports have identified SphK, S1P, and S1P receptors as key modulators of pulmonary diseases [225,226], with S1P/S1PR signalling linked to pulmonary inflammation caused by viral infections, such as the influenza virus [217,227,228]. The mortality of patients with the H5N1 strain of influenza was associated with high pharyngeal virus loads and hypercytokinemia [229]. Within the lung, S1PR_1_ is expressed on the endothelial cells and lymphocytes [230]. In infectious diseases, S1PR_1_ influences recruitment and the trafficking of innate immune cells, macrophage polarisation, and plasmacytoid dendritic cell functions [117]. Extracellular S1P binding to S1PR_1_ also plays a dual role in inflammation by the activation of intracellular inflammatory signalling pathways during viral infections. During influenza (H1N1) infection, S1PR_1_ agonists protected mice, and ferrets against acute immunopathologic damage in influenza infections [217,227,228]. Using an endothelial cell-specific inducible S1PR_1_ knockout mouse line (*S1PR_1_-ECKO*), S1PR_1_ gene ablation resulted in an increased inflammatory reaction with the aggravation of lung injury, massive exudation, and highly oedematous, with vascular haemorrhaging and a significant increase in inflammatory cell infiltration, in response to H_1_N_1_ influenza virus challenge [217]. Heightened levels of responsive cytokines/chemokines in *S1PR_1_-ECKO* were also observed, indicating an aberrant pulmonary immune response. In the study by Teijaro et al., S1P/S1PR_1_ was shown to mediate two signalling pathways involved in inflammatory reactions, MAPK and NFkB, in H1N1-infected mice [230]. Teijaro et al. demonstrated that S1PR_1_ agonists suppress cytokines and innate immune cell recruitment, thus inhibiting early proinflammatory cytokine expression and innate immune cell build-up, which blunts the cytokine storm [230]. Activation of S1PR_1_ using a S1PR_1_ agonist, CYM-5442, was protective against viral challenge in control mice, but not in the *S1PR_1_-ECKO* mice, highlighting the importance of S1PR_1_ signalling in the control of viral infection. Thus, selectively targeting S1P_1_ receptors, as central orchestrators of cytokine storm suppression, may prove to be promising in alleviating the severity of diseases where amplification of the cytokine storm is a significant pathological manifestation [230], such as demonstrated in acute and chronic COVID-19 patients. As such, S1P analogues have been successful in reducing lung tissue inflammation and injury in response to viral diseases in preclinical mouse models [231]. Conversely, as briefly mentioned in Section 5.3, intracellular SphK-S1P signalling has been demonstrated to regulate LPS-induced inflammation in lung endothelium, whereby LPSs have been very effective in the activation of signals to induce an antiviral response [129].

### 5.7. SphK/S1P/S1PRs in Maintaining Vascular Integrity

Catastrophic vascular endothelial failure is a distinctive feature emerging from severe SARS-CoV-2 infection, as described in the postmortems of COVID-19 patients [6,232]. Loss of vascular endothelial integrity underlies the major severe COVID-19 symptoms of hyperinflammation, oedema, and tissue ischaemia. The S1P-S1PR signalling pathway is crucial in maintaining vascular endothelial integrity. Abnormalities in this signalling network lead to devastating consequences, morbidity, and death. Most of our understanding of the roles of S1P signalling in the vasculature is derived from in vivo mouse models and in vitro primary human umbilical vein endothelial cells (HUVEC) [17,233,234,235,236]. Mice deficient in SphK1 and SphK2 die prematurely from haemorrhages, resulting from a dysfunction in vascular development [122]. SphKs play vital roles in vascular function both by enabling and secreting the active form of sphingosine (S1P) essential in vascular formation (angiogenesis and vasculogenesis), blood pressure homeostasis, barrier protection and integrity, and vascular tone (mainly through SIPRs_1-3_) [110,237,238]. S1P is also important in the migration and differentiation of endothelial cells lining the inside wall of the blood vessels. Pathophysiology of the endothelial cells plays a vital role in lung disorders, cardiovascular diseases, and heart failures, such as impaired coronary development and systemic perfusion, and has a major impact on morbidity and death [57].

Red blood cells, platelets, fibroblasts, and vascular endothelium are rich sources of plasma-S1P, whereby S1P acts as a pleiotropic lipid mediator critical for the regulation of vascular and immune cells through activation of its cognate receptors (SIPR_1-3_) [239]. In addition, lymphatic endothelial cells are the main source of lymph secreted S1P [240].

In blood and lymph vessels, S1P concentrations are naturally high, and conversely, S1P expression is low in cells and tissues due to the higher activity of S1P dephosphorylation enzymes (endoplasmic reticulum-resident S1P lyase) inside the cells [240]. High blood S1P levels are essential for S1P functions, including blood vessel integrity, and recruitment of inflammatory cells [115]. In addition, S1P signalling has a central role in the regulation of lymphocyte trafficking whereby the S1P gradient needs to be maintained between the systemic circulation and tissues for egress of newly formed T cells from the thymus, and movement of mature T and B cells from secondary lymphoid organs [241,242].

Plasma-S1P levels are tightly regulated, having a rapid turnover of approximately 15 min. S1P is constitutively produced by SphK1 and SphK2 (sphingosine to S1P) and degraded by S1P lyase, specific phosphatases (SPP1 and 2), and lysophospholipid phosphatase 2 (LL3) [17,243]. SphK1 and SphK2 both contribute to S1P extracellular egress to maintain plasma-S1P levels [244]. Deletion of either SphK isozyme, in vivo and in vitro, demonstrate complementary, compensatory, as well as distinct functions in maintaining plasma-S1P levels and vascular endothelial integrity [233]. The continuous supply of S1P from the endothelial cells contributes significantly to vascular integrity and homeostasis, through stabilisation of endothelial adherens junctions and prevention of microvessel leakage [17,243]. As previously mentioned, (HDL)-apolipoprotein M (ApoM) and (Albumin-S1P) have different functional properties [208,209]. Christoffersen et al. demonstrated circulating plasma-SIP bound to HDL-Apo M (ApoM) is important for basal endothelial function and vascular protection [209]. S1P-signalling was found to be more stable when bound to HDL-ApoM compared to albumin-S1P. In the absence of HDL-ApoM-S1P, in ApoM-null mice, the endothelial cell barrier function was impaired, even in the presence of albumin-S1P [208]. Albumin-S1P, as opposed to HDL-ApoM-S1P, exhibits differential effects on the trafficking and stabilisation of S1PR_1_ signalling [208]. This highlights a significant role for HDL-ApoM-S1P in maintaining vascular homeostasis. During inflammation, activation of SphK1-dependent S1P, or inflammation-induced cell death releasing active SphK2 into the extracellular space, helps to maintain extravascular S1P levels and vascular integrity [245].

In addition, secreted S1P can be protective against chronic inflammation, as demonstrated in animal models whereby during acute and chronic inflammation plasma-S1P limits disruption of the vascular endothelial layer and reduces oedema [246]. Endothelial defects can be rectified in some cases by increases in plasma HDL-ApoM-S1P binding to S1PR_1_ to induce adherens junctions and vascular integrity, thereby protecting the vascular barriers. HDL-ApoM-S1P also suppresses proinflammatory cytokine signalling and angiogenic signals, such as VEGF growth signalling, to maintain vascular integrity [115]. Thus, maintaining plasma-HDL-ApoM-S1P levels may be beneficial in suppressing inflammation, sepsis, and other pathological conditions [115].

#### Differential Roles of S1P_1-3_ Receptors in Vascular Function and Regulation

Of all the S1P receptors, only S1PR_1-3_ are expressed in the vascular endothelial cells, S1PR_1_ being the most abundant [247]. S1PR_1_ is the most well-studied S1P receptor as it is ubiquitously expressed in innate immunity, mediating functions in most innate immune cells [117]. The removal of S1PR_1_ (Edg1) in mouse models is embryonic-lethal and has some compensatory functions for S1PR_2-3_ [234,248]. S1PR_1_ blocks the formation of new blood vessels (a negative regulator of angiogenesis), regulates cellular adhesion and motility (strengthens the adherens junctions), and restricts sprouting angiogenesis through regulation of the vascular endothelial growth factor 2 (VEGFR2) signalling and internalisation [235]. The deletion of S1PR_1_ (S1PR_1_ null mice) results in disorganisation of the aorta and endothelial hyperplasia, vascular leakage, exaggerated and ectopic endothelial sprouting and embryonic lethality [235,247]. Activation of S1PR_1_ prompts the release and circulation of new blood platelets to prevent blood loss during injury, and mice lacking S1PR_1_ develop severe blood clotting (thrombocytopenia) [236]. S1PR_2_ and S1PR_3_ expressed in the vascular endothelial, albeit in lower amounts compared to S1PR_1_, have some redundant and compensatory functions, as well as independent functions in endothelial development via the Gi signalling pathway (Figure 1) [114]. One recognised role of S1PR_2_ is to oppose the activity of S1PR_1_. S1PR_2_ has been shown to repel rather than attract cells in response to S1PR_1_ [249]. The deletion of all three S1P receptors in S1PR_1-3_ null mice demonstrate a more severe vascular phenotype than S1PR_1_ null. Individual S1PR_2_-null, or S1PR_3_-null, demonstrate no overt adverse phenotype; however, double-null S1PR_2_ and S1PR_3_ show partial embryonic lethality and vascular development [114].

Further studies characterising the three S1P receptors (S1PR_1-3_) functions demonstrate differential roles for the receptors in homeostasis and disease. As reported by Zhao et al., the inhibition of S1P lyase increased S1P levels in lung tissue and bronchoalveolar lavage fluids, resulted in reduced lung injury, by stemming alveolar flooding, and inflammation, providing some endothelial barrier protection [250]. Therefore, injection of the S1P lyase inhibitor to maintain S1P levels in the blood has been suggested as a potential therapy to maintain vascular endothelial barrier function. However, the full story proves to be not so simple. This barrier-protective effect was only demonstrated by S1P binding to S1PR_1_, not S1PR_2_ or S1PR_3_ [17]. When S1PR_2_ and S1PR_3_ were activated by S1P, disruption was observed in the alveolar and vascular barriers, with increased permeability, and conversely, blocking these receptors was found to be beneficial [251,252]. S1P receptor agonists and antagonists have been developed to block one or more of the S1PR receptors (Figure 1).

### 5.8. SphK/S1P/S1PR_1_ Role in Thrombosis

One of the unusual and life-threatening side effects in COVID-19 patients is unusual blood clotting, believed to occur as a direct action of the virus on the arteries themselves, resulting in uncommon strokes in younger patients, pulmonary embolisms, immune complications, and multiorgan failure [10]. The commonly used blood thinners for the dissemination of blood clots are not reliable and young and old patients are dying [10]. As early as 1957, sphingosine was shown to be associated with the prevention of blood clot formation [253]. Blood platelets are the body’s natural defence to prevent excessive bleeding in the event of blood vessel damage, are involved in vascular wall repair, and are likely key effector cells in immune and inflammation response to infection [254]. Conversely, blood platelets are heavily involved in the process of arterial thrombosis [243]. Platelets express large amounts of SphK which produce and secrete abundant amounts of S1P when activated [243]. They lack the S1P lyase enzyme, which degrades S1P; therefore, platelets can store a ready source of S1P in response to platelet activation [243].

S1P/S1PR_1_ activation is associated with the haemostasis-related mechanisms of the coagulation system [255]. One of the many proposed roles of S1P in the blood, through its activation of S1PR_1_, is a master regulator of efficient thrombopoiesis, as demonstrated in mice where severe thrombocytopenia was observed in a mouse model lacking S1PR_1_, and activation of the S1P/ S1PR_1_ signalling promoted the release of new platelets into the bloodstream [236]. Biological effects of S1P release in the vasculature include the prevention of platelet aggregation, and thrombosis-related vascular diseases [255,256]. Upon vascular injury, thrombus formation and stabilisation require persistent platelet recruitment and activation. S1P-secretion from human platelets is stimulated by thrombin (thrombin induces SphK expression to produce S1P), S1P in turn, activates S1PR_1_ and G_i_-dependent activation of Rac-1 signalling, via cross-talk with endothelial protein C receptor, and one of the beneficial consequences of this later action is to limit and counteract thrombin-induced endothelial damage [257]. Thus, thrombin both enhances endothelial generation of S1P and also limits its own action through differential regulation of S1P/S1PR signalling pathways. The integrity of thrombus formation was shown to be dependent on SphK2 activation of S1P in a mouse model, where deficiency in SphK2 resulted in defective platelet aggregation and arterial thrombosis [258].

### 5.9. SphK/S1P/S1PR and Sepsis

Potentially life-threatening sepsis is a major complication in COVID-19 patients. Sepsis is characterised by a hyperinflammatory systemic response, reducing the body’s ability to deal with opportunistic bacterial infection in the bloodstream, and triggering changes that can damage multiple organ systems. Sepsis-related mortality is linked to endothelial dysfunction and microvascular thrombosis underlying multiple organ failure. Although we have a limited understanding of the connection between S1P and sepsis, serum levels of S1P, especially HDL-ApoM-S1P are compromised in septic patients and are inversely associated with sepsis disease severity [254,259,260]. Sepsis is also associated with increased S1PR_2_, whereby S1PR_2_ positivity is associated with increased severity of sepsis, and deficiency of S1PR_2_ in a sepsis mouse model improved bacterial clearance and survival [261]. S1PR_2_ also modulates endotoxin-induced inflammation in the endothelium [259]. In experimental sepsis in mice, impaired function of the heart was improved by administration of FTY720, or by deletion of SphK2, which in both cases resulted in increased serum-S1P levels, and preservation of cardiac function [260]. S1P is a potent regulator of endothelial integrity; therefore, a reduction in the levels of S1P may contribute to sepsis-induced organ failure by promoting capillary leakage and impaired tissue perfusion contributing to opportunistic bacterial invasion and colonisation.

### 5.10. SphK/S1P/S1PR and Cardioprotection

Long-term or permanent heart damage is one of the extreme symptoms of COVID-19. There is a broad consensus that S1P signalling plays a critical role in cardioprotection, by maintaining cardiac cell survival and function [262,263]. Receptors for S1P are present in cardiac vascular endothelial and smooth muscle cells, as well as cardiac fibroblasts [262,263]. The S1P receptors are involved in the remodelling, differentiation, and proliferation of cardiac fibroblasts (mainly S1PR_3_) [262]. S1PR knockout studies in mice strongly suggest that both S1PR_2_ and S1PR_3_ mediate cardioprotection from ischaemia/reperfusion injury in vivo [262]. Conversely, significant increases in cardiac S1P, SphK1 and S1PR_1_ are observed in postmyocardial infarction (MI) associated with the proinflammatory response, and inhibition of this inflammatory pathway may benefit patients with MI [263].

### 5.11. SphK/S1P in Neuroinflammation and Neurodegeneration

Thromboembolic events are becoming frequently observed characteristics of COVID-19, with young people presenting with cerebral venous system thrombosis [64,65]. SphK/S1P regulates all the different brain cell populations and is involved in most fundamental cell processes, including neural development and survival. The role of SphK/S1P in the brain is complex and has been likened to a “double-edged sword” in the brain [264]. SphK/S1P mediate vital signalling pathways involved in the infiltration of peripheral immune cells in the CNS during neuroinflammation; however, the protective immune response can quickly change into chronic neuroinflammation and can lead to neurodegeneration, impaired cognition, and synaptic losses [265,266]. S1PR_1_ and S1PR_2_ are expressed in the blood vessels of the brain where they mediate distinguishable cellular responses during an acute ischaemic event. S1PR_1_ activation protects blood–brain-barrier (BBB) function and attenuates inflammation whilst most studies suggest that S1PR_2_ promotes BBB loss of integrity and elicits a proinflammatory phenotype [267]. Thus, selective S1PR1 ligands, particularly at the cerebrovascular level, are gaining an appreciation as effective modulators of stroke pathogenesis due to the ability to preserve BBB integrity and to attenuate the development of vascular inflammation [268]. The recent probable association between SARS-CoV-2 infection and Parkinson’s disease has triggered the notion that COVID-19 patients have an increased risk of developing Parkinson’s disease later in life and possibly other long-term neurological disorders [67]. Recently reported, a positive neuroprotective effect of S1PR modulators (SEW2871 and FTY720, Table 1) in a preclinical mouse model, demonstrated reduced neuroinflammation and prevention of Parkinson’s disease symptoms [147], further supporting ongoing S1P-therapy in the prevention of chronic COVID-19 neurological dysfunction.

## 6. Repurposing Anti-SphK-S1P-S1PR Compounds in Curtailing COVID-19 Symptoms

### 6.1. FTY720 in the Prevention of SARS-CoV-2 Infection and Therapy for COVID-19 Patients

The repositioning or repurposing of existing drugs to help reduce COVID-19 symptoms helps to fast-track therapy. Toxicity studies, safe drug dosage, routes of administration, and adverse outcomes are currently available for many drugs. Fingolimod (FTY720, Gilenya^®^, Novartis-2-amino-2-[2-(4-octylphenyl)ethyl]propane-1,3-(diol)), a first-in-class S1PR immunomodulator, used successfully in multiple sclerosis, is now in clinical trials as a potential adjuvant therapy for COVID-19 patients (Clinicaltrials.gov Identifier: NCT04280588). As one of the best-characterised sphingolipid-based drugs, it is a prime candidate for COVID-19 treatment. Many of the studies supporting the use of this candidate modulator are based on both in vitro (cell culture) and in vivo (animal, and a vast accumulation of human clinical data) data. As with most drugs, the outcome is very much dependent on the dosage and timing of drug administration.

FTY720 is a sphingosine analogue and, like sphingosine, requires SphK for phosphorylation and activation, and is, therefore, classified as a prodrug. FTY720 acts as a substrate and is transformed into its active state, FTY720-P, by the action of SphK (SphK1 and SphK2) isozymes, with a much higher affinity for SphK2 (30-fold more efficient), compared to SphK1 [269]. Activated FTY720 (FTY720-P) acts paradoxically as both an agonist and antagonist. FTY720 is an unselected agonist for four of the five G-protein-coupled-S1PRs, namely S1PR_1,3,4,5_ (Figure 1). However, unlike the naturally produced S1P, FTY720-P also acts as a selective antagonist for S1PR_1_ by specifically inducing S1PR_1_ internalisation and downregulation [266,270]. In this way, the intracellular S1PR_1_-mediated signalling pathway is desensitised (see Section 5.4, The S1P/S1PR Paracrine “Outside–Inside” Response).

One of the potential positive effects of FTY720-P antagonism of S1PR_1_ in COVID-19 therapy is the curbing of hyperinflammation or “the cytokine storm”. Lymphocytes normally circulate between the blood (high levels of S1P) and the lymphoid tissue (low levels of S1P) as regulated through the S1P gradient whereby S1P binds to S1PR_1,3,4,5_ receptors on the lymphocytes signalling them to travel to the lymph [115]. The binding of FTY720-P to the S1PR_1_ on lymphocytes leads first to activation and then subsequent S1PR_1_ downregulation (i.e., by the internalisation and degradation of S1PR_1_ on the lymphocytes), thus preventing aggressive infiltration of lymphocytes from the lymphoid tissue into the blood, bringing about a state of peripheral lymphopenia, and limiting the inflammatory response [115]. Thus, FTY720-P can limit blood vessel damage caused by excessive inflammation.

S1P/S1PR_1-3_ is essential for maintaining vascular integrity. Similarly, FTY720, and a selective S1PR_1_ agonist, AUY954 (Figure 1, Table 1), have been shown to reduce microvascular permeability as well as inflammation in animal models [246]. Conversely, two specific S1PR_1_ antagonists, W146 and NIBR-0213 (Figure 1 and Table 1), demonstrated loss of capillary integrity in animal models [146,271].

These combined results essentially imply that the agonist effect of the S1P-mimetics may be useful in suppressing damage to blood vessels and preserving blood vessel barrier integrity, whilst the antagonist effects of FTY720-P reduce inflammation by reducing aggressive lymphocyte egress. In contrast to most immunosuppressants, FTY720 sequestration of lymphocytes in the lymphoid tissue appears not to cause cytotoxicity [178,272].

The S1P_1_ receptors have been likened to the central orchestrators of cytokine storm suppression in pathogenesis [230], therefore, a secondary effect of FTY720 is through desensitisation of intracellular S1PR pathways by acting as a functional antagonist, thus acting to deactivate the potential inflammatory S1PR intracellular pathways [217,227,228] (also see Section 3.1, The S1P/S1PR Paracrine “Outside–Inside” Response).

The FTY720 prodrug can pass through the blood–brain barrier (BBB) and exert several direct effects in the CNS, from neuroprotection to reduction of neuroinflammation. As an approved FDA-drug, FTY720 may help in early intervention and prevention of the more severe neurological side effects of COVID-19. One such example is FTY720-P inhibition of aggressive egress of lymphocytes to the CNS by lymphocyte confinement in the lymphoid tissues, thus limiting neuroinflammation [266] (see Section 5.11 SphK/S1P in Neuroinflammation and Neurodegeneration). A second example is the potential of reduction of blood clots in the brain underlying the causation of strokes in younger COVID-19 patients [10].

Approximately 20% of COVID-19 patients experience cardiovascular symptoms, with approximately 40% of deaths related to cardiovascular complications [14,40,49,50,51]. As mentioned, maintenance of S1P/S1PR signalling plays an important and protective role against cardiovascular endothelial dysfunction; however, overstimulation of the inflammatory response by S1P/S1PR_1_ is also a major factor in heart disease and heart failure. Blocking the S1P/S1PR_1_-mediated inflammatory response by FTY720 administration has been shown to exhibit positive effects against heart disease [57], and, as observed in ischaemia and hypoxic injury mouse models, it has been proven to be protective against acute and chronic myocardial injury [263,273].

Other potential beneficial effects of FTY720 agonism on S1PR_1,3,4,5_ in COVID-19 treatment include the regulation of thrombopoiesis through S1PR_4_ activation. Severe SARS-CoV-2 infections can lead to sepsis, a fatal side effect in COVID-19 patients, mediated by inflammation-elicited endothelial barrier leakage and cytokine release; however, this can be alleviated by FTY720 [274].

On a note of caution, prolonged treatment has been shown to compromise barrier function, increasing permeability and vasculature leakage in cell and mouse models [115,275,276,277]. Controversially, FTY720 increases lymphopenia, which is associated with a poorer prognosis and death, especially in younger COVID-19 patients [278,279]. Nonetheless, the beneficial effects of FTY720 on hyperinflammation, vascular integrity, thrombosis, sepsis, and heart disease associated with severe COVID-19, as discussed in this review, could alleviate the worsening symptoms associated with these severe COVID-19 cases. Hence, timing and dosage of FTY720 are important, “too much of a good thing can turn bad”.

Whilst FTY720 is a prime candidate for COVID-19 adjunct therapy, due to its proven safety and efficacy as an immunomodulator, there are potential detriments with continuous use and overuse of this drug. New generations of S1PR agonist and antagonist drugs have/are being developed and some are currently undergoing clinical trials. An updated list can be viewed in Table 1 [115,133,141,183,280].

In the past two years, two additional selective S1PR immunomodulatory drugs have been FDA-approved. In March 2019, Mayzent (Siponimod, Novartis) was approved, and in March 2020, Ozanimod. Both drugs are agonists for S1PR_1_ and S1PR_5_.

### 6.2. Ozanimod—A safer COVID-19 Alternative S1PR Therapy

Recently, Ozanimod (RPC1063, molecular formula, C_23_H_24_N_4_O_3_, trade name Zeposia^®^), a more specific S1PR agonist drug, targeting two SIP receptors (S1PR_1,5_) [281], has received FDA approval (March 2020) as an immune modulator [282]. Similarly to FTY720, binding of Ozanimod to S1PR_1,5_ leads to the internalisation and degradation of the S1P receptors and a reduction in circulating lymphocytes. Unlike FTY720-P, Ozanimod does not bind and activate S1PR_3_ [281]. This may confer a drug advantage in the clinic, as Ozanimod does not demonstrate cardiac conduction abnormalities or hypertension, and there was no evidence of fibrosis observed in the clinical use of FTY720 [281]. Having a much shorter half-life, Ozanimod (t_1/2_ is 19 h in humans), compared to FTY720 (t_1/2_ is 24–25 h in humans), and with a much shorter lymphocyte recovery (Ozanimod = three days compared to FTY720 = 4–8 weeks), confers some advantages including flexibility of treatment with other immune modulators if complications do occur with S1PR-targeted treatment. Two examples where the rapid cessation of immune modulators was imperative are provided. The first is the occurrence of opportunistic infections, where treatment with FTY720 was linked to several cases of opportunistic fungal infections where, if left untreated, could have caused severe fungal meningitis, as cited [117]. In a second example S1PR_1_ inhibitors are known teratogens and, therefore, cannot be used during pregnancy. In this case, the washout period is much longer with FTY720 compared to Ozanimod [281].

### 6.3. Opaganib—A SphK2 Specific Inhibitor in COVID-19 Therapy

Recently, Opaganib (ABC294640, 3-(4-chlorophenyl)-adamantane-1-carboxylic acid (pyridin-4-ylmethyl) amide, or trade name Yeliva^®^) [133,196], a specific SphK2 inhibitor, has been listed to start global randomised phase II/III clinical trials for COVID-19 patients (ClinicalTrials.gov Identifier: NCT04467840), and a United States randomised phase II study (ClinicalTrials.gov Identifier: NCT04414618). Opaganib is a sphingosine mimetic, which competitively binds SphK2, thus preventing phosphorylation of sphingosine to its active form S1P and, therefore, effectively reducing intracellular levels of S1P and reducing intracellular signalling-induced inflammatory pathways [200].

SphK2 is a critical host factor in viral replication, supporting a conceivable role in the replication-transcriptional complex of positive single-stranded RNA viruses. Therefore, blocking SphK2 using Opaganib reduces both viral reproduction and minimises the potential risk of resistance due to viral mutation development [200].

An intriguing attribute of SphK2 is that blocking SphK2 leads to increased circulating S1P levels in mice (three times the normal level), whereas blocking SphK1 results in decreased levels of circulating S1P (approximately half the normal level) [133]. As circulating S1P is important in vascular integrity, there is some validity in opanganib therapy, on the one hand, reducing the viral load by inhibiting viral replication and pathological inflammation, whilst, on the other hand, increasing S1P production through SphK1 activation to maintain vascular endothelial integrity.

One of the perceived negative downsides of using SphK2 inhibitors is that SphK2 deletion increases inflammatory cytokine production and macrophage activation [132], which may contribute to detrimental side effects for COVID-19 patients. However, in preclinical studies, Opaganib has demonstrated anti-inflammatory properties against pathological inflammation, decreased fatality in an influenza virus mouse model, and improved *Pseudomonas aeruginosa*-induced lung injury [214]. In a recently completed clinical trial for “compassionate use” (ClinicalTrials.gov Identifier: NCT04435106), in a small cohort of Opaganib treated COVID-19 patients with severe symptoms, the drug was safe and well tolerated, with clinical and laboratory improvement in all patients.

## 7. Concluding Remarks

Effective innate and adaptive inflammatory immune responses are important in curtailing virus infection. When the virus has invaded the host cell, it is difficult to eradicate and the body’s immune system may overreact (i.e., a hyperinflammatory response), as demonstrated effectively in the case of many COVID-19 patients. A unifying theme underlying most of the COVID-19 symptoms is the loss of vascular integrity affecting the major organs of the body, including the lungs, heart, and brain. Specific targeting of components of the SphK/S1P/S1PRs signalling pathway can subvert many of the severe complications of COVID-19, including (a) reduction of the hyperinflammatory response (cytokine storm) whilst preserving vascular endothelial integrity, which is seemingly a major route of COVID-19 maliciousness in multiorgan failure (specifically protection against pulmonary, neurological and cardiovascular symptoms), (b) reduction/prevention of blood disorders such as thrombotic complications (clot formation), (c) attenuation of sepsis and importantly, (d) overall averting rapid clinical COVID-19 patient deterioration.

We started writing this review based on a question, “can modulating the SphK-S1P-S1PR pathway reduce severe COVID-19 disease symptoms?” From historical and contemporary data on the involvement of the sphingolipid pathway in viral infections, inflammation, and vascular integrity there is strong support demonstrating a role for modulating components of the SphK-S1P-S1PR in COVID-19 disease management. The immunomodulatory properties of the sphingolipids produce a plethora of beneficial effects, not only in curtailing SARS-CoV-2 infectivity and treatment of COVID-19, but also for the ongoing management of the acute and chronic severe side effects of COVID-19. To date, three S1P-based FDA-approved drugs, FTY720, Ozanimod, and Opaganib are being repurposed for COVID-19 treatment and are currently in clinical trials highlighting the potential for targeting the SphK-S1P-S1PRs to reduce COVID-19 symptoms. However, more specific agonist and antagonist drugs targeted to individual and multiple S1P receptors have been developed (Table 1 and Table 2), firstly, to explore the complex biological signalling and function of the SphK isozymes and the five S1PRs (G-protein-coupled transmembrane receptors), and, secondly, to assess their potential as therapeutics. S1P-S1PR targeting, alongside antiviral treatment, may prove to be beneficial in the prevention of COVID-19 deaths and the control of future coronavirus infections.

## Figures and Tables

**Table 1:** Comparative selectivity of the S1P modulators.

S1P Modulator	S1PR Selectivity	References
**Agonists**		
# FTY720 *# Fingolimod and phosphorylated fingolimod (Trade name: Gilenya)	S1P_1_ > S1P_5_ > S1P_4_ > S1P_3_	[127,135,136,137,138,139]
S1P-specific antibody	Depletion of S1P	[112]
*# CS-0777	S1P_1_ > S1P_5_ > S1P_3_	[140]
* Ponesimod (ACT-128800)	S1P_1_ > S1P_5_ > S1P_3_	[141]
*# Ozanimod (RPC1063)	S1P_1_ > S1P_5_	[141]
* Ceralifimod (ONO-4641)	S1P_1_>S1P_5_>S1P_4_	[142,143]
* Siponimod (BAF312)	S1P_1_ > S1P_5_ > S1P_4_	[144]
* GSK2018682	S1P_1_ > S1P_5_	[141]
SEW2871	S1P_1_	[145,146,147]
AUY954	S1P_1_	[148,149]
* Amiselimod (MT-1303)	S1P_1_, S1P_4_, S1P_5_	[141]
* Etrasimod (APD334)	S1P_1_, S1P_4_, S1P_5_	[150]
* ASP4058	S1P_1_, S1P_5_	[151,152]
* Mocravimod (KRP-203)	S1P_1_>S1P_4_	[153,154]
AAL(R) and phosphorylated AAL(R) (FTY720 analogue)	S1PR1, S1PR3, S1PR4 S1PR5	[155,156,157]
CYM-5442	S1P_1_	[158,159]
VPC23153	S1P_4_	[160,161]
W-061	S1P_1_ > S1P_5_ > S1P_4_ > S1P_3_	[142,162]
* Cenerimod	S1P_1_	[163]
# CYM-5478	S1P_2_	[164]
SB649146	S1P_1_	[165,166,167]
Antagonists		
VPC44116 VPC23019 VPC25239	S1P_1_ and/or S1P_3_	[112] [168]
TASP0277308	S1P_1_	[169]
** Sonepcizumab (Mab)	S1P_1_	[170]
W146	S1P_1_	[171,172]
JTE-013	S1P_2_	[173,174]
NIBR-0213	S1P_1_	[171]

Note: Adapted from [102]. More in-depth reviews [112,134,175,176,177]. * Currently in clinical trials, refer to [178] for more details. # agonists known to act as functional antagonists. ** Sonepcizumab—monoclonal antibody (Mab) binds to S1P and prevents S1P/S1PR interaction.

**Table 2:** Sphingosine kinase (SphK) inhibitors.

SphK Inhibitor	SphK Selectivity	References
SKi (2-(p-hydroxyanilino)- 4-(p-chlorophenyl)thiazole) or SK1-II	SphK1 and SphK2	[112,179,180]
Safingol	SphK1 and SphK2	[181]
L-threo-dihydrosphingosine (DHS)	SphK1 and SphK2	[182]
N,N-dimethyl-D-erythro-sphingosine (DMS)	SphK1 and SphK2	[112]
B-5354c, F-12509A (Natural products)	SphK1 and SphK2	[112]
ABC294735	SphK1 and SphK2	[179]
Amgen 82	SphK1 and SphK2	[183]
Amidine-based range of sphingosine analogues	SphK1 and SphK2	[112]
MP-A08	SphK1 and SphK2	[141]
ST-1083	SphK1 and SphK2	[184]
S-15183a and S-15183b (Natural product)	Not specified	[112]
SKI-V	Noncompetitive?	[185]
*PF-543 ((R)-(1-(4-((3-methyl-5-(phenylsulfonylmethyl)phenoxy)* methyl)benzyl)pyrrolidin-2-yl)methanol), SK1-5c (CAY10621), SK1-178, VPC96091 (36a), CB5468139	SphK1	[186] [180,187]
SKI-I	SphK1	[188,189,190]
LCL351	SphK1	[191]
Compound inhibitors 51 and 54	SphK1	[141,192]
Balanocarpol	SphK1	[193]
VPC94075	SphK1	[157]
1-deoxysphinganines 55-21 and 77-7 (induces proteasomal degradation -SK1)	SphK1	[194]
RB-005	SphK1	[195]
(S)-FTY720 vinylphosphonate	SphK1	[196]
Genzyme	SphK1	[183,197]
Peretinoin (NIK333)	SphK1	[198,199]
ABC294640	SphK2	[112,200]
SG-12 and SG14 (sphingosine analogue)	SphK2	[201]
SLC5111312 and SLM6041434	SphK2	[202]
F02 thiourea adduct of sphinganine	SphK2	[194]
VT-ME6	SphK2	[203]
(2S,3S,4R)-Pachastrissamine	SphK2	[204]
Trans-12a and Trans-12b	SphK2	[203]
SLR080811, SLP120701	SphK2	[180]
K145	SphK2	[180]

Adapted from [205].
